# Supplementary material for: Human–Animal Interactions with Bos taurus Cattle and Their Impacts on On-Farm Safety: A Systematic Review
Source: Animals (Basel). 2022 Mar 19;12(6):776. doi: 10.3390/ani12060776 (PMC8944486; doi:10.3390/ani12060776)
Supplement: Supplementary file 1 [file animals-12-00776-s001.zip › animals-1622604-supplementary.pdf]

Supplementary Table S1: Summary of papers included

Authors, Year and Origin	Type and Domain of Study	Animal Origin	Breed	Animal Type	Sample Size (n)	Measurement of Exposure and Outcome	Aim	Findings and Recommendations	Limitations and Critical Appraisal
Algueperse and Vassuer 2021 (Quebec, Canada)	Primary data, observational study	Dairy	Holstein	Cows	47 (16 summer, 16 winter, 15 autumn) 3 x 2 factorial	Human reactivity test (cows reaction to a strange person's approach)and suddenness test (cows' reactivity to a sudden event)	To study the impact of regular, outdoor, exercise provision in different seasons on reactivity of tie stall cows	Outdoor access and handling method impacts cow reactions	A protocol for moving cattle was provided, however there was seasonal variation in the handling systems used due to weather which may have had a confounding effect.
Alwall et al., 2019 (Sweden)	Primary data, survey	Not defined	Not defined	Not defined	23 Farmers (5 women, 18 men; age range 34- 75 years)	Farmer descriptions of safety practices on farm, themes included 1- concerns for work safety in the context of production 2-occupancy 3- organisation 4- daily operations 5- barriers the perceived to improving farm safety 6- steps taken to improve farm safety	To develop an understanding of what the farmers perceived as a risk and subsequent actions to remove threat.	Simple, common and direct threats will lead to adaptive threat reducing behaviour whereas complicated general and indirect threats promoted maladaptive behaviour which reduced fear but did not reduce threat	Survey was piloted to assess suitability and this resulted in updates to make it more accessible to farmers. Semi-structured interview followed a 6 point guide. Study limited as it only covered a small geographical area, farmers had previously taken part in a safety scheme.

Coetzee et al., 2010 (US)	Survey of vets who castrate calves in the USA	Not defined	Not defined	Bull calves	189 Bovine veterinarians	Veterinarians methods for castration and reasons chosen	Describe castration methods and adverse events and husbandry of US vets at the time of castration	Respondents identified: Potential risk to operator (70%) , calf size (71%), handling facilities (62%), and experience (61%) as either critically or very important when choosing a castration method. Further research needed into castration practices to optimise animal and handler welfare.	Aims clearly stated and target population identified as veterinarians. Survey piloted on veterinarians prior to dissemination. 1,972 vets contacted through professional bodies, 189 responses received (9.2% response rate), the low response rate was reported to be typical of this population. Research focussed on USA
Destrez, et al., 2018 (Burgundy, France)	Primary data, observational study and questionnaire	Beef	Not defined	Cows	20 Farms, 40 - 160 cattle	Semi structured interview to understand the human animal relationship, Questionnaire to assess farmer attitude and an avoidance test to evaluate herd reactions to approach from an unfamiliar human	Investigate the relationship between herd management, attitude to their animals and handling and animals reactions to humans	Farmers practices linked to avoidance distance, Monitoring cattle frequently, including behaviour in genetic selection and physical contact during monitoring can reduce animal avoidance distance	Small sample size (20 farms) which varied in size. Farmers selected through beef consultants, and were known to be interested in understanding cattle behaviour. Semi structured interview had 3 points presented in open ended format, and questionnaire was 42 questions

Ebinghaus, et al., 2018 (Middle and Northern Germany)	Primary data, observational study and questionnaire	Dairy	Predominantly Holstein (>=50%)	Cows	32 farms, two winter periods , herd size 29 - 530 (mean 102.7 sd 106.8)	Avoidance distance, tolerance to tactile interaction, behaviour during release from restraint, qualities behaviour assessment of animal throughout study, stock persons attitude through a questionnaire, herd, housing and management characteristics potentially related to handling, and herd, housing and further management characteristics	Investigated possible effects of stockperson and farm related factors on cows´ behaviour towards humans in different test situations	Positive attitude, frequent human contact can positively affect a cows disposition	Multiple measures of cow behaviour and a Majority of farms sampled (24) were organic and may not be representative of all management systems.
Estevez-Moreno et al 2021 (Spain)	Primary data, focus group discussions	Beef	Pyrenean	Cattle (all)	37 male farmers aged between 25 and 65 years old distributed in 4 focus groups (n 8,9,11,9)	Farmer description of temperament of pyre nan breed, comparison with other breeds, anatomical behavioural indicators, ontogenic and phylogenic indicators of behaviour	Identify the temperament related traits of the Pyrenean cattle, genetic factors impacting temperament and how farmers interpret temperament	Participants thought animal sensory acuity was linked to temperament, external features less reliable indicators of temperament. Farmers identified differences between sexes and individual animals within breed.	The participants were selected using an inclusion criteria and were not aware of the scientific study of temperament and were all active farmers involved working directly with cattle. Moderator ensured all participants were involved and followed a semi structures guide. Limited comparison to other breeds and farmers may have different interpretations/ descriptions of certain behavioural terms

Fraser-Williams et al., 2016	Secondary data, Scoping review (International literature search, UK media and guidelines search)	Not defined	Not defined	Cattle (all)	Published literature (n=8) Media reports (n=89)	Number of incidents of walkers attacked by cattle, and safety recommendations	To assess available information about negative interactions between the public and cattle and to identify rick factors for cattle attacks and evaluate current guidelines	Members of the public amongst cattle must be careful around cattle. The risk of attack may be greater in the presence of dogs, persons' actions Recommended mitigations varied greatly from different sources.	Records were media reports published after the incident and did not have detailed reports of human behaviour and facilities involved in incident. Included so the records assessed were not complete.
Kullolli et al., 2017 (India)	Secondary, review of hospital records in Indian hospital	Not defined	Not defined	Bull	Hospital records of 15 bull attacks reviewed	Inpatient particulars, date of admission, discharge date, location of main would, surgery required. Data grouped by injury location	Summarise bull induced injuries to prepare for treatment	Bull gore injurious are commonly encountered in rural India, and surgeons should be prepared.	The items reviewed were hospital records reported after the incident and did not have detailed reports of human behaviour and facilities involved in incident. the paper does not explain why the patients were handling the bulls, why did the bulls become aggressive for no apparent reason
Kutzer et al., 2015 (Switzerland, Leichenstien)	Primary data, Observational study	Dairy	Brown Swiss, Holstein Friesian and brown Swiss	Heifers	72	Animal behaviour at milking, cardiac activity, avoidance distance and milk yield	To assess if training a primiparous heifer could improve behaviour when milking	Trained heifers showed fewer signs of stress at milking, however this was not reflected in cardiac activity	Aim stated. The first milking for each animal was not observed, so may have been recorded on the first milking observed (between first and third) was recorded. This was evenly distributed amongst farms
Lange et al., 2020 (Trenthorst, Germany)	Primary data, Observational study	Dairy	Not defined	Cows	36, 3 treatment groups: gentle interactions when: tied stall; free in barn; or no additional interactions	Avoidance and approach behaviour of cows	To improve animal human relationship so that the animal is more receptive to touch	Animal in both the control and free groups had improved relationship with humans, but not significantly changed in tied stall gentle interactions should take place when the animal is unrestrained	Aim stated clearly and controlled experiment balanced by lactation number.

Liebman, et al., 2016 (Wisconsin, US)	Primary data focus group discussions	Dairy	Not defined	Cattle (all)	37 immigrant workers from Wisconsin dairy farms (23 men and 14 women)	Worker reported experience on farm	To qualitatively describe the knowledge, attitudes and practices of immigrant dairy workers. Examined themes of worker injury, worker compensation perception of hazards and hazard abatement	Workers find animals in general potentially hazardous with bulls, primiparous heifers and freshly calved cows most unpredictable. immigrant workers less likely to report incident as they feared losing their job	Focus groups were carried out in the immigrants native language, with moderators trained in focus group methods and fully bilingual. Audio recordings transcribed, and where not possible notes were taken independently by two scribes and merged. Findings were anonymised, however one focus group was carried out on farm which had more positive findings. Qualitative data may not be representative.
Lindahl et al., 2015 (Sweden)	Primary data, Observational study and Farmer survey	Dairy	Not defined	Cows	12 farms; mean 157.5 cows (range 45-430); 12 Handlers; mean age 36.8 years; (range 23 – 64)	Handlers' stress, handlers' demographics, handlers' attitude to cows, time spent in the risk zone, Physical contact which could have caused injury	To gain an understanding of how stress, handler attitudes and behaviour affect risk and safety during handling of dairy cows	Time spent in risk zone increased physical contact from cow, no other significant effects	Same dataset with different analysis that Lindahl (2016) Small dataset meaning some results may have been due to chance. Used same trained individuals at each farm to reduce subjectivity.
Lindahl et al. 2016, (Sweden)	Primary data, Observational study and Farmer survey	Dairy	Not defined	Cows	12 farms; mean 157.5 cows (range 45-430); 12 Handlers; mean age 36.8 years; (range 23 – 64)	Moving cows to adverse (hoof trimming) and less averse (Milking) procedures, Physical contact which could have caused injury; cow heart rate	To gain an understanding of how stress, handler attitudes and behaviour affect risk and safety during handling of dairy cows	Moving cows to hoof trimming elicited a greater deviation in heart rate than moving to milking (indicating more stress on animal).Positive stock person attitude towards animals and frequent positive handling are associated with better human animal relationships.	Aim stated. Behavioural measurements and cardiac measurements taken, however only 3 cows had cardiac measurements taken, and 3 different cows were measured for each treatment. Selection process for participants not stated. Small sample size

Sheldon et al., 2009 (Global)	Secondary data, Scoping review	Not defined	Not defined	Bull	Internet search and search of American Agricultural databases; 287 cases examined	Date of incident, name of victim, location, gender, age, experience of victim with handling bulls, nature of injury, nature of incident, fatal/nonfatal, site, breed/species, size, and final outcome of bull	To examine bull induced injury reports and find links between incidents	Review farm infrastructure. Victims may be overconfident with youths neither strong nor mature enough to manage bulls. Recommends aggressive bulls should be slaughtered, consider genetic tests. The authors decided that there was not enough data on age of bull, type and size etc. to make an unbiased statement on the most dangerous bull.	The study identifies a lack of comprehensive data on bull attacks. The items reviewed were often records after the fact and may not have had detailed reports of human behaviour and facilities involved in incident
Simon et al., 2016 (California, US)	Primary data, Observational study and Farmer survey	Beef	Not defined	Cows	30 herds, 3065 cattle(102 +- 40 observations per ranch range 28 - 182)	Cattle behaviour - Balking, running, stumbling/ falling within chute, vocalization; Handling Tail twist, moving aid , cattle prod, miscatch; facility design and farmer attitude	The objectives of this study were to measure cow–calf health and handling welfare outcomes and gather management, facility, and producer perspective information to 1) describe current practices and 2) inform assessment design.	Opportunities to improve stock person handling and cattle behaviour. Facilities differ across ranches and are not always aligned with scientific or industry recommendations.	Farmers selected though extension officers and so may have been more progressive than other ranchers.

Turner et al., 2013 (Scotland)	Primary data, observational study	Beef	Mixed beef	Heifers, cows	Farm 1. 143 cows (crossbred Limousin, n = 58; crossbred Aberdeen Angus, n = 52; purebred Charolais, n = 22; purebred Luing, n = 11) (mean age 5.2 yr ±SD = 2.97) Farm 2. 309 Limousin crossbred cows (mean age 6.4 yr ±SD = 2.03)	Cow precalving temperament using crush score, flight speed and isolation score; Post calving defensiveness assessed when stockperson handled calf for tagging or moving cow calf pair for isolation, Cow maternal behaviour recorded through time lapse video of cow calf interaction, including feeding time and cow proximity to calf,	To estimate the repeatability of and correlation between cow temperament traits and how maternal behavioural traits impact progeny productivity	Precalving temperament is repeatable within parity and crush score and postcalving temperament repeatable across parities. Precalving temperament and postcalving defensiveness are largely independent. Some correlation between cow fear and progeny's productive traits.	The results are precise and confidence intervals are given, however only two farms over two years were recorded so repeatability limited. Different measures for variables used on each farm so difficult to directly compare. Limited because only 2 farms were used in this study
Volling 2012 (Lower Saxony, Germany)	Primary data, survey	Dairy	Holstein, Deutsches schwarzbuntes niederungsrind	Cows	85 organic farmers	Farmer description of polling and cattle behaviour on farm	To find out numbers of polled cattle and reasons for polling organic cattle and if farmer is willing to stop.	Main reason for dehorning fear of injuries to animals or farmers. Majority saw problems with horns and plan to move to genetically polled cattle	Survey carried out on a high proportion (82%) of organic farmers in a small target area. Set questions were asked and the farmer provided freeform answers. Limited by an inadequate discussion on whether dehorning should be banned. This study was a conference paper rather than journal article.
